# Psychometric properties of Drug Attitude Inventory among patients with schizophrenia

**DOI:** 10.4102/sajpsychiatry.v28i0.1760

**Published:** 2022-04-29

**Authors:** Oladipo A. Sowunmi

**Affiliations:** 1 Department Of Clinical Services, Neuropsychiatric Hospital Aro, Abeokuta, Nigeria

**Keywords:** Drug Attitude Inventory, schizophrenia, validation, Aro, Nigeria

## Abstract

**Background:**

The treatment of patients diagnosed with schizophrenia has remained three-fold physical (pharmacological), psychological and social. Furthermore, the need to monitor adherence to the physical aspect of treatment has been a major concern to mental health practitioners as this usually affects the success of psychological and social treatment.

**Aim:**

My study aimed to determine the psychometric properties of Drug Attitude Inventory (DAI) among patients with schizophrenia. The study was carried out at the Neuropsychiatric Hospital, Aro Abeokuta Ogun State and on an average, about 150 patients were seen daily at the outpatient clinic.

**Methods:**

Internal consistency, item-total correlation (the two-way mixed method with absolute agreement) and Cronbach’s alpha were evaluated using an intra-class correlation coefficient (ICC). This instrument’s level of adequacy was determined using factor analysis (principal component analysis with varimax rotation).

**Result:**

Marital status and level of education were significantly associated with adherence. The Cronbach’s alpha was 0.56 and principal components factor analysis with varimax rotation produced a three-factor solution.

**Conclusion:**

My study has shown that the DAI is a valid and reliable instrument and can be used in a clinical setting where there are limitations with time such as the outpatient clinic.

## Introduction

Schizophrenia has been regarded as one of the major psychiatric disorders with psychosis as its major feature.^1,2,3^ It may be considered as a disorder that affects the way a patient thinks and perceives which then influences the patients’ behaviour and his affective response to his environment. Due to this effect on mood and behaviour, science has been on the search for a cure and made tremendous progress with the discovery of chlorpromazine in the 1950s.^1,2,3^

While this may be considered a breakthrough, the treatment of patients diagnosed with schizophrenia has remained three-fold physical (pharmacological), psychological and social.^4^ Furthermore, the need to monitor adherence to the physical aspect of treatment has been a major concern to mental health practitioners as this usually affects the success of psychological and social treatment. Many methods have been proposed most with their merit and demerit.^5,6^ However, very few have looked at how the psychological and social components of a patient’s state of mind influence compliance to antipsychotic medication intake, the so-called behavioural toxicity, dysphoric subjective response and psychophysiological sensitivity.^5,6^

In simple terms, one of the key components of antipsychotic medication compliance evaluation is to measure patients’ attitude to antipsychotic drug-taking. This attitude to antipsychotic medication compliance may reflect subjective complaints (which include extrapyramidal or autonomic side effects, or these may be associated with akathisia or pharmacologically related affective blunting) which may influence sustained adherence. Moreover, it is important to note that side effect is not biased to either compliant or non-compliant patients, it is their subjective attitude and interpretation of their medicated state that ultimately determine whether or not they will be adherent to antipsychotic medication use.^5,6^

One of the instruments which can be used to evaluate the attitude of patients diagnosed with schizophrenia and who are managed with antipsychotic medication is the Drug Attitude Inventory (DAI) English Version which has not been previously evaluated in Nigeria. My study aims to present (1) the psychometric properties of this instrument among patients with schizophrenia in a Nigerian setting and (2) compare with the values previously reported while showing the relationship between compliance or non-compliance and sociodemographic characteristics of these patients.

## Methodology

### Study site

I carried out this study at the Neuropsychiatric Hospital, Aro Abeokuta Ogun State. This is a Federal Government owned psychiatric hospital established in 1954. It is a specialist tertiary institution located along the Lagos Abeokuta Road near Ita-Oshin, Abeokuta. Although the hospital has a nationwide catchment area, the majority (89%) of its patients are from South Western Nigeria.^7^ This hospital is staffed by psychiatrists, psychiatrists – in – training, mental health nurses, social workers and Clinical Psychologists. It provides inpatient, outpatient and 24-h emergency services to mentally ill patients and patients with neuropsychiatric conditions. It has a total capacity of 546 beds for inpatient care, 153 beds at the main hospital and 393 beds at the Lantoro annexe. Patients who have been discharged from inpatient care, as well as those who were never admitted, are seen at the outpatient clinic (OPC). Those who are managed primarily as outpatients are usually first assessed at the emergency or assessment unit of the hospital after which they continue with follow-up treatment at the OPC. On an average, about 150 patients are seen daily at the outpatient clinic.^4^

### Sample size determination

The total sample size in my study was 220 and the calculation of the sample size and oversampling was done as proposed by Cochrane using the subject to item ratio method (*n* = 20).^8^ From the hospital, medical records of 54 patients diagnosed with schizophrenia were seen on each clinic day.

### The instrument

This questionnaire was devised by Awad et al., while it was later modified into Hogan’s DAI of 30 items. The number of items was later decreased to 10 from 30 items with ‘yes’ or ‘no’ questions. Six of the answers are expected to be affirmative, while four are expected to be negative to implicate drug compliance. Patients receiving total score points greater than zero are considered drug compliant, while those equal to or less than zero are considered drug non-compliant.^9^ The studies on the psychometric properties of the original English version show that it is a reliable and valid scale.^10^ Internal consistency (Cronbach’s alpha = 0.81) as well as test-retest reliability (intra-class correlation coefficient [ICC] = 0.82) was high. In addition, the correlations between this instrument and the rating of medication influences (ROMI) measure were 0.56 for the ‘reasons for compliance’ scale and –0.47 for the ‘reasons for non-compliance’; and the correlation between DAI and the Neuroleptic Dysphoria Scale15 was 0.76 before antipsychotic medication was begun and 0.74 at 48 h of having taken it. Hogan et al. (1983 and 1992) demonstrated that DAI is capable to predict treatment compliance in patients with schizophrenia and the response to treatment with antipsychotics (measured with BPRS: *r* = –0.75; and with GAF: *r* = 0.68).

### Study design with inclusion and exclusion criteria

This is a study that I conducted to evaluate the psychometric properties of DAI and data were collected in 2018 over 3 months. Respondents were recruited by me using a systematic random sampling of every 4th patient diagnosed with schizophrenia (using International Classification of Disease Version 10 [ICD-10] at first contact in the hospital and reconfirmed with the psychotic Module of Mini International Neuropsychiatric Interview English Plus Version 5 [MINI PLUS]) registered to be seen at the OPC of the hospital. A total of 220 respondents with a diagnosis of schizophrenia, aged 18–64 years and above and who were able to read and write in English were recruited.

Exclusion criteria included patients who were not mentally stable enough to participate in my study. This was determined with the psychotic module of MINI PLUS: any patient with symptoms suggested by the clinical judgment section of psychosis M8b, M9b, M10b such that interview became impossible. Patients with other general medical and mental comorbidities were also excluded. Although (response rate was 100% as no one declined to participate) more than 220 participants were approached in my study, participants with incompletely filled questionnaires were eliminated at the final stage of analysis. Thus, approximately 8.3% of them were excluded. Ethical approval was obtained from the research and ethics committee of the hospital and permission was obtained from the managing consultants. Verbal and written informed consent was obtained from all participants and caregivers, mental fitness was sought from the managing consultants and the attention of the managing consultants was drawn to their corresponding patients who had problems with medication adherence.

### Data analysis

Data were analysed using the Statistical Package for Social Science (SPSS version 23) Computer Software. The level of significance was set at *p* ≤ 0.05. The ICC was used to determine the internal consistency of the instrument and Cronbach’s alpha for the entire scale. Factor analysis using principal component analysis with varimax rotation was used to confirm the robustness of the original construct. The item-total correlation was done with the ICC using the two-way mixed method with absolute agreement.

### Ethical considerations

Ethical approval was obtained from the research and ethics committee of the hospital and permission was obtained from the managing consultants. Verbal and written informed consent was obtained from all participants and caregivers, mental fitness was sought from the managing consultants and the attention of the managing consultants was drawn to their corresponding patients who had problems with medication adherence.

## Result

Table 1 shows the frequency of response of each of the items of the DAI questionnaire. The mean total scale score of respondents in my study was 4.49 ± 3.92. Other details are highlighted in Table 1.

**TABLE 1:** Mean scale score and frequency of Drug Attitude Inventory item scale of respondents (*n* = 220).

Number	Questions	Response	*n*	%
1	For me, the good things about medication outweigh the bad	False	60	27.3
		True	160	72.7
2	I feel strange, ‘doped up’, on medication	True	61	27.7
		False	159	72.3
3	I take medications of my own free choice	False	85	38.6
		True	135	61.4
4	Medications make me feel more relaxed	False	29	13.2
		True	191	86.8
5	Medication makes me feel tired and sluggish	False	72	32.7
		True	148	67.3
6	I take medication only when I feel ill	False	83	37.7
		True	137	62.3
7	I feel more normal on medication	False	32	14.5
		True	188	85.5
8	It is unnatural for my mind and body to be controlled by medications	False	98	44.5
		True	122	55.5
9	My thoughts are clearer on medication	False	44	20.0
		True	176	80.0
10	Taking medication will prevent me from having a breakdown	False	42	19.1
		True	178	80.9

Prevalence of Adherence (DAI Score): Non-Adherence = 39 (17.7); Adherence = 181 (82.3).

DAI Score total (mean) = 4.49 ± 3.92.

Table 2 shows the association between sociodemographic variables and adherence. Marital status (χ^2^ = 10.69; degrees of freedom (*df*) = 1; *p* ≤ 0.01) and level of education (χ^2^ = 6.59; *df* = 2; *p* = 0.03) were observed to be significantly associated with adherence. Other details are depicted in Table 2.

**TABLE 2:** Association between sociodemographic variables and compliance.

Variables	DAI adherence indicator				Statistics		
	Non-adherence		Adherence	
	*N*	%	*N*	%	χ^2^	df	*p*
**Gender**	-	-	-	-	0.33	1	0.56
Male	19	19.4	79	80.6	-	-	-
Female	20	16.4	102	83.6	-	-	-
**Marital status**	-	-	-	-	10.69	1	< 0.01
With partner	08	8.2	89	91.8	-	-	-
Without partner	31	25.2	92	74.8	-	-	-
**Level of education**	-	-	-	-	6.59	2	0.03
No formal education or primary education	04	7.1	52	92.9	-	-	-
Secondary education	21	23.9	67	76.1	-	-	-
Tertiary education	14	18.4	62	81.6	-	-	-
**Ethnicity**	-	-	-	-	1.87	1	0.17
Yoruba	33	16.6	166	83.4	-	-	-
Igbo, Hausa, or Others	06	28.6	15	71.4	-	-	-
**Religion**	-	-	-	-	0.19	1	0.66
Christianity	30	18.4	133	81.6	-	-	-
Islam or Traditional African Religion	09	15.8	48	84.2	-	-	-

DAI, Drug Attitude Inventory

The Cronbach’s alpha was 0.56 (Cronbach’s alpha if item deleted was not significant for any of the 10-item analysed) with an intra-class correlation (ICC) of 0.55 and a *p*-value of < 0.001 in my study. Further details can be found in Table 3.

**TABLE 3:** Cronbach’s alpha and intra-class correlation coefficient of the Drug Attitude Inventory.

Cronbach’s alpha	Cronbach’s alpha based on standardised items	*N* of items	Intra-class correlation coefficient †	95% Confidence interval		*F* test with true Value 0			
				Lower bound	Upper bound	Value	*df* 1	*df* 2	*p*
**Reliability statistics**									
0.56	0.58	10	-	-	-	-	-	-	-
**Intra-class correlation coefficient**									
Single measures	-	-	0.11‡	0.077	0.146	2.286	219	1971	< 0.01
Average Measures	-	-	0.55§	0.454	0.630	2.286	219	1971	< 0.01

A two-way mixed-effects model where people effects are random and measures effects are fixed.

† , Type A intra-class correlation coefficients using an absolute agreement definition.

‡ , The estimator is the same, whether the interaction effect is present or not.

§ , This estimate is computed assuming that the interaction effect is absent because it is not estimable otherwise.

A principal component factor analysis with varimax rotation, retaining factors with an Eigenvalue greater than 1, produced a three-factor solution. See Table 4. After rotation, factor 1 (items 1, 4, 7, 9 and 10) accounted for 22.90% of the variance, factor 2 (items 2, 5 and 8) accounted for 12.99% of the variance and factor 3 (3 and 6) accounted for 12.00% of the variance. Thus, in total, the rotated factor solution accounted for 48.44% of the total variance.

**TABLE 4:** Rotated component matrix of 10-item Drug Attitude Inventory.

Rotated component matrix†	Component		
	1	2	3
DAI-10 test Q7	0.727	0.136	0.021
DAI-10 test Q9	0.668	0.120	−0.280
DAI-10 test Q10	0.667	−0.046	0.202
DAI-10 test Q4	0.596	0.111	0.004
DAI-10 test Q1	0.311	−0.028	−0.159
DAI-10 test Q2	−0.175	0.779	−0.060
DAI-10 test Q8	0.171	0.704	−0.064
DAI-10 test Q5	0.247	0.525	0.283
DAI-10 test Q6	0.087	0.205	0.772
DAI-10 test Q3	0.255	0.257	−0.645

DAI, Drug Attitude Inventory.

Extraction Method: Principal Component Analysis.

Rotation Method: Varimax with Kaiser Normalisation.

† , Rotation converged in five iterations.

## Discussion

The multifariousness of factors involved in adherence problems requires appropriate matching if interventions in people diagnosed with schizophrenia are expected to be optimal.^9,11,12,13^ Sociodemographic attributes are reported to be both definitive and non-definitive for drug compliance in patients. However, my finding was in support of sociodemographic variable been definitive.^9,11,12,13^ I observed that those with partners and primary level of education were significantly associated with adherence to antipsychotic medication. This was, however, contrary to what was reported in two previous studies in Turkey and Sweden^9,13^ where no significant associations were reported. Cumulative suggestions appear to tilt towards the fact that whether or not there will be a difference may be related to the large sample size, the level of functioning of the patients investigated and their symptom severity.^9,11,13,14^ In the main study, having a partner was a significant factor that emphasised the importance of social support in the war against non-adherence. While one would have expected that the higher the level of education, the better the compliance would be. This was not the case in my study and it suggests that medication adherence may not be directly related to cognitive prowess before the onset of illness but on cognitive stability after the onset of illness which may be associated with the client’s subjective feelings to the burden of illness, perception of side effect and insight to illness.^9,11,13,14^

In addition, I observed that the drug attitude was reliable with a moderate-to-high Cronbach’s alpha; this finding was similar (0.57) to what was reported in the Swedish version^13^ of the same instrument which further supports a reliable instrument across race. A three-factor component was observed in my study, which is similar to previous reports in Turkey and Sweden.^9,13^ Factor 1 which was made up of questions 1, 4, 7, 9 and 10 was like the factor named ‘Comments on the protective effect of Medication’. Our factor 2 which was made up of questions 2, 5 and 8 depicted the factor named ‘Comments on side effects of Medication’ whilst our factor 3 which was made up of questions 3 and 6 represented the factor named ‘Positive effect of drugs’. This is in keeping with earlier studies and supports the fact that DAI is a reliable and valid instrument.

## Strengths and limitations

My study is the first study to report the psychometric properties of DAI in Nigeria. However, my study is not without limitations. Firstly, an evaluation of the psychopathology rating and insight rating was not done at the onset of my study which would have provided more information on the adherence pattern observed in my study. Secondly, a cross-sectional study cannot confirm a causal association between the sociodemographic factors and compliance with antipsychotic medication use. Next, future studies should assess participants’ capacity to consent to participate in the study using specific instruments that may provide great improvement. Moreover, attitude towards adherence was measured solely based on subjective assessments and was not controlled through objective methods. Furthermore, the categorical approach to analysing DAI-10 scores could limit the finding of additional correlations. Finally, another limitation was the lack of assessment of additional factors related to attitude towards medication, such as therapeutic alliance, duration of untreated psychosis and type of pharmacological class used in the treatment.

## Conclusion

In conclusion, I am able to show that DAI is a valid and reliable adherence instrument within the community it was evaluated. Future studies should access capacity, insight while evaluating adherence attitude. The role of education remains a subject to be investigated fully. Be that as it may, my study shows that it is important to make policies and plan towards the negative medication attitudes in patients diagnosed with schizophrenia as it appears to be a complex obstacle hindering compliance in these patients. Drug attitude together with other clinical predictors which are identified to be related to non-adherence, can indicate when patients are going to need adherence promoting strategies, through motivational interviewing, alliance enhancing methods, pill counts and computerised reminders.
